# Effects of high-protein concentrates with different rumen undegradable-to-degradable protein ratios on performance and carcass traits in 26-month-slaughtered Hanwoo steers

**DOI:** 10.5713/ab.24.0728

**Published:** 2025-02-27

**Authors:** Woo Hyeong Hong, Rajaraman Bharanidharan, Tae Hoon Kim, Jun Suk Byun, Geum Hwi Bang, Krishnaraj Thirugnanasambantham, Joonpyo Oh, Ridha Ibidhi, Sun Sik Jang, Kyoung Hoon Kim

**Affiliations:** 1 Department of International Agricultural Technology, Graduate School of International Agricultural Technology, Seoul National University, Pyeongchang, Korea; 2 Department of Eco-friendly Livestock Science, Institutes of Green Bio Science and Technology, Seoul National University, Pyeongchang, Korea; 3 Department of Geography, McGill University, Montreal, QC, Canada; 4 University of Maryland Center for Environmental Science, Frostburg, MD, USA; 5 Farmsco Co., Ltd., Anseong, Korea; 6 Pondicherry Centre for Biological Sciences, Puducherry, India; 7 Cargill Animal Nutrition Korea, Seongnam, Korea; 8 INRAE, Institut Agro, Univ. Bourgogne Franche-Comté, Agroécologie, Dijon, France; 9 Hanwoo Research Institute, National Institute of Animal Science, Pyeongchang, Korea

**Keywords:** Dietary Protein Level, Hanwoo, Rumen Undegradable Protein, Shortened Fattening Period

## Abstract

**Objective:**

This study investigated the effects of high-protein concentrates with two different rumen undegradable protein (RUP)-to-degradable protein ratios on the performance and carcass characteristics of Hanwoo steers reared for 26 months.

**Methods:**

Thirty-two Hanwoo steers (217±13 kg) were randomly allocated to eight pens (four animals/pen) and assigned to one of two concentrate treatments (four pens/treatment), low-RUP concentrate (LRUP; RUP ratio of 37:63) or high-RUP concentrate (HRUP; RUP ratio of 47:53), with a dietary crude protein (CP) content of 20% dry matter (DM) during the growing stage (8 to 17 months) and 18% DM during the fattening stage (18 to 26 months).

**Results:**

Increasing the RUP ratio in the concentrate reduced (p = 0.062) rumen ammonia (NH3-N) levels during the growing stage. A decrease (p<0.05) in the molar proportions of acetate and valerate, and an increase (p = 0.097) in the proportions of iso-valerate, was observed in the HRUP group. Significant improvements (p<0.05) in the average daily gain and the feed conversion ratio were observed in the HRUP group during the fattening stage. Although there were no significant group differences in carcass yield or characteristics in steers slaughtered at 26 months, a trend (p = 0.081) toward an increased rib-eye area was observed in the HRUP group. Relative mRNA expression profiling revealed higher lipid biosynthesis and lipolysis in the HRUP group at slaughter. However, no effects on intramuscular fat content was detected.

**Conclusion:**

Raising the RUP level improved performance and intramuscular fat metabolism in Hanwoo steers. The final body weight of the steers in the HRUP group was comparable to that of Hanwoo steers raised under the conventional 30-month fattening period. Overall, feeding a high-CP concentrate with a higher RUP proportion (47% CP) may be a beneficial strategy to shorten the feeding period.

## INTRODUCTION

The feeding period for Hanwoo steers has changed over the last two decades. Hanwoo steers had previously been fed for 24 months, which was extended to 30 months to produce sufficient intramuscular fat (IMF) [1,2]. The feeding program in the majority of commercial beef cattle farms in Korea is based on a diet with high dietary crude protein (CP) content that decreases gradually; 18% to 19% of total dry matter intake (DMI) in the growing stage (9 to 13 months of age), 16% to 18% DMI in the fattening stage (14 to 21 months of age), and 14% to 16% DMI in finishing (22 to 30 months of age) stage [3]. Dietary energy content was gradually increased to supply sufficient energy for stimulating fat deposition during the 17-month fattening stage [3]. However, a long fattening period can lead to reduced energy efficiency and increase the inedible fat in beef. In addition, it is associated with higher production costs and lower net profits due to higher international grain prices, as feed ingredients rely entirely on imports.

Attempts have been made in Korea to shorten the feeding period of Hanwoo steers by increasing the dietary level of total digestible nutrients (TDNs), including CP [4,5]. Oh et al [6] reported a 13% increase in the average daily gain (ADG) of Hanwoo steers fed a high-CP diet (21% dry matter [DM]) compared to a low-CP diet (15% DM) at the fattening stage (15 to 22 months of age). Similarly, Bharanidharan et al [5] reported a relatively lower lipid turnover rate in Hanwoo steers fed a high-CP diet (17% DM) compared to a low-CP diet (14.5% DM) at the fattening stage (18 to 23 months of age), which may favor shortening of the feeding period. However, growing cattle, unlike mature animals, require higher quantities and improved quality (in terms of degradability) of dietary CP to meet their elevated amino acid demands for muscle accretion. Excessive protein degradation in the rumen during the fattening stage can negatively impact energy flow due to the high energy expenditures associated with ammonia (NH_3_) detoxification [7]. Therefore, precision nutrition strategies that improve nitrogen (N) availability without compromising energy efficiency are essential for maximizing productivity in beef cattle.

Soybean meal (SBM), the most commonly used protein source in cattle diets, provides a rich supply of rumen degradable protein (RDP) and a well-balanced amino acid profile. However, it has certain limitations, including low rumen undegradable protein (RUP) levels and an imbalanced methionine to lysine ratio [8]. To address this, heat processing followed by fermentation can significantly increase the RUP content in SBM, resulting in fermented soybean meal (FSBM) [9]. Unlike RDP, which is broken down by rumen microbes and can lead to inefficient nitrogen utilization, RUP bypasses ruminal degradation and is digested post-ruminally, enhancing the absorption of metabolizable protein in the small intestine and ultimately improving animal performance [10]. Improvements in RUP levels have been linked to enhanced marbling scores (MS) in Hanwoo steers [11]. Consequently, optimizing the RUP-to-RDP ratio in a high-CP diet by adjusting SBM and FSBM levels may offer additional benefits, including a potential reduction in the feeding period. However, studies investigating the effects of high dietary CP and RUP levels on the performance and carcass characteristics of Hanwoo steers are limited [5,11–13]. In particular, few studies have examined the effects of high CP and RUP diets initiated during the growing stage of Hanwoo steers [11]. Moreover, this is the first study to provide benchmark data for a deeper understanding of the growth patterns and carcass characteristics of Hanwoo steers raised over a short feeding period of 26 months. We hypothesized that increasing N availability by increasing the supply of RUP through inclusion of FSBM from the growing stage onward might increase N use efficiency and improve the performance of Hanwoo steers, potentially yielding results comparable to a 30-month feeding program. Therefore, this experiment aimed at investigating the effects of high CP concentrates with two different RUP levels, fed at the same energy level from the growing to the late fattening stage (8 to 26 months of age), on growth rates, rumen fermentation, carcass characteristics, and the intramuscular expression of genes related to fat metabolism in Hanwoo steers slaughtered at 26 months.

## MATERIALS AND METHODS

### Experimental design, animals, and diet

All experiments were carried out at the animal farm of Seoul National University (Pyeongchang, Korea). The methods and protocols for all animal experiments were approved by the Institutional Animal Care and Use Committee of Seoul National University (SNU-210615-2) and were performed following the relevant guidelines and regulations. After an adaptation period of 1 month to the barn and the basal diet, 32 Hanwoo steers with an average initial live weight of 217±13 kg (average age = 8 months) were randomly assigned to one of two concentrate treatments: SBM-based concentrate with a low RUP:RDP ratio (LRUP; 37:63 of total CP) and FSBM-based concentrate with a high RUP:RDP ratio (HRUP: 47:53 of total CP). The FSBM used in this study was SoELAB-Pass, provided by FEEDUP Ltd., Korea. The steers were grouped into pens (n = 4 steers/pen), resulting in four pen replicates per treatment. Growing (8 to 17 months of age) and fattening (18 to 26 months of age) stages were distinguished. The ingredients and chemical composition of the diet fed during the growing and fattening stages are shown in Tables 1, 2, respectively. The target total CP concentration for both concentrate formulations was set at 20% DM for the growing stage and 18% DM for the fattening stage. While the measured CP concentration in the concentrates fed during the growing stage was close to the target, the CP concentration in the LRUP (20% DM) was higher than that in the HRUP (17.8% DM) during the fattening stage (Table 2). The TDN and energy density levels remained the same for both groups during both stages. Timothy hay and rice straw hay were offered as a forage source at the growing and fattening stages, respectively. All animals were fed following the feed manufacturer’s feeding program. The DMI was restricted, and the amount fed was consistent between the groups throughout the experiment until slaughter. The animals were fed twice daily (09:00 and 17:00) and were restrained for 2 h, using self-locking stanchions to consume their own diet. Feed refusals were recorded for 3 consecutive days per 10-day interval, and DMI was calculated. Animals were allowed free access to water and a mineral block. Body weight (BW) was measured once monthly during the entire experiment period. The feed conversion ratio (FCR) was calculated as total DMI divided by total BW gain.

### Rumen fluid, blood, and tissue biopsy sampling

Rumen fluid and blood samples were collected from all steers at 15 months of age. Approximately 200 mL of rumen fluid was collected from each steer 2 hours after the morning feeding using a stomach tube connected to a vacuum pump (Oriental Dream, Hwaseong, Korea). The first 200 mL of fluid was discarded to prevent contamination with saliva. The rumen fluid was immediately filtered through four layers of muslin, and the pH was measured using a pH meter (model AG8603 Seven Compact pH/Ion S220; Mettler-Toledo, Schwerzenbach, Switzerland). The samples were stored at −20°C for further analysis of NH_3_-N and volatile fatty acid (VFA) concentrations, as described previously [14].

Blood samples were collected 3 hours after the morning feeding via jugular venipuncture using an 18-G syringe and transferred to anticoagulant-free 8.5 mL yellow-capped BD Vacutainer SST II advance tubes. The samples were centrifuged at 3,000×g for 10 min (1580R; Labogene, Seoul, Korea), and the serum was transferred to 2 mL microtubes for storage at −80°C until further analysis of the serum biochemical parameters using an automatic blood analyzer (BS-400; Mindray, Beijing, China).

*Longissimus lumborum* (LM) muscle tissue samples (2 g/steer) were obtained at 17 months of age from the left side of the third lumbar vertebra in eight steers per treatment group (two steers per treatment per pen) using a spring-loaded biopsy instrument (Biotech, Košice, Slovakia), under intramuscular sedation (Xylazine 20 Inj., 20 mg/steer; KEPRO B.V, Deventer, Netherlands) and a local anesthesia line block injection (10 mL/steer, 2% lidocaine injection; Cheil Pharma, Seoul, Korea). The LM muscle samples were immediately frozen in liquid nitrogen and stored at −80°C until analysis. The animals were intramuscularly injected with procaine penicillin G (4500 IU/kg, G.C. GPS Inj.; Green Cross Veterinary Products, Seoul, Korea) immediately after the biopsy. Additionally, the animals were intramuscularly administered 3 mg/kg ketoprofen (New-Procop Inj.; Shinil Biogen, Yesan, Korea) for 3 consecutive days.

### Carcass evaluation, sampling, and image analysis of marbling fleck characteristics

All steers were transported to a local slaughterhouse at 26 months of age. They were provided with *ad libitum* water but no feed for approximately 12 h before slaughter. On the following day, the animals were slaughtered according to the Korean regulations for animal care and standard procedures following the ethical guidelines for animal welfare. After 24 h of chilling, the carcasses were assessed for yield and quality grade by an official grader, following the Korean Carcass Grading Procedure.

After chilling for 24 h, a 1-cm-thick muscle slice was collected between the 12th and 13th right thoracic vertebrae (*longissimus thoracis* [LT]) of the cold carcasses, free of subcutaneous fat and connective tissue, from all 32 steers. The slices were homogenously ground through a 5-mm plate using a grinder (MGB-32; Hankook Fujee Industries Co. Ltd., Suwon, Korea) and snap-frozen in liquid nitrogen for further analysis of the fatty acid composition and relative mRNA levels of the lipid metabolic genes.

Digital images of the LT sections were captured using a mirror-type camera (HK-333; HayasakaRikoh Co. Ltd., Sapporo, Japan) to analyze marbling fleck characteristics with Beef Analyzer II software (Hayasaka Ricoh Co. Ltd.), as described previously [3].

### RNA extraction and real-time quantitative polymerase chain reaction

RNA was isolated from the LM and LT muscle samples using the RNeasy Lipid Tissue Mini Kit (Qiagen, Hilden, Germany) following the manufacturer’s instructions. After assessing the quality and integrity of the RNA, cDNA was synthesized using the PrimeScript 1st Strand cDNA Synthesis Kit (Takara, Shiga, Japan) according to the manufacturer’s protocol. Real-time polymerase chain reaction (PCR) analysis to quantify the relative mRNA levels of genes involved in fat metabolism was performed using SYBR Green Real-Time PCR Master Mix (Bioneer, Daejeon, Korea), following the methods and primers described in Bharanidharan et al [3].

### Chemical and fatty acid composition analyses

Feed samples were dried in a forced-air oven at 65°C for 72 h to calculate the DM content, and ground samples passed through a 1-mm screen (Model 4; Thomas Scientific, Parsippany, NJ, USA) were obtained for analyzing chemical composition, as described in Bharanidharan et al [3].

The intramuscular lipid content of the LT muscle samples and the fatty acid composition of the LM and LT samples were determined using methods described previously [3].

### Statistical analysis

The Shapiro-Wilk test was performed to check the normality of the data. When the data were not normally distributed, they were transformed using the PROC RANK procedure in SAS 9.4 (SAS Institute, Cary, NC, USA). The analysis of rumen fluid characteristics, blood metabolites, fatty acid composition, relative mRNA expression levels, carcass characteristics, and animal performance was performed using the PROC MIXED procedure in SAS 9.4. The pen was used as the experimental unit, and the model included treatment as the fixed effect. Individual animal data were included in the model, with a random effect of pen and animal nested within treatment. LSMEANS was used to calculate the means. A p-value <0.05 was considered significant.

## RESULTS

### Intake, performance, and carcass characteristics of Hanwoo steers slaughtered at 26 months

No significant differences (p>0.05) in the concentrate or total DM intake were observed between the dietary treatments throughout the experiment (Table 3). However, a higher (p<0.001) CP intake was detected in the LRUP group during the fattening stage due to the greater measured CP concentration in the formulated concentrate. Additionally, RUP intake was higher (p<0.01) in the HRUP group during both stages. While there were no differences in initial or final BW, higher (p<0.05) total weight gain and ADG were observed in the HRUP group. A lower (p<0.05) FCR was observed in the HRUP group than in the LRUP group during the fattening stage.

The differences in the dietary RUP ratio did not significantly affect carcass or marbling fleck characteristics (Table 4). However, there was a tendency toward an increase (p = 0.081) in the rib-eye area and a decrease (p = 0.085) in dressing percentage in the HRUP group.

### Rumen fermentation and blood metabolite profile of Hanwoo steers at the growing stage

Increasing the RUP ratio did not significantly affect (p>0.05) ruminal pH or total VFA production (Table 5). However, a decrease (p<0.05) in the molar proportions of acetate and valerate was detected, and a trend toward an increase (p = 0.097) in iso-valerate proportions was observed in the HRUP group. Additionally, a decrease (p = 0.062) in rumen NH_3_-N concentration was detected in the HRUP group. No significant differences (p > 0.05) were observed in blood urea nitrogen (BUN) or other blood metabolites between the groups (Table 6).

### Intramuscular fatty acid composition of muscle biopsy tissue and carcass longissimus muscle

The IMF content and fatty acid composition of the LM and LT tissues are presented in Tables 7, 8, respectively. Increasing the RUP ratio did not significantly affect the intramuscular fatty acid content of the Hanwoo steers during either the growing stage (LM) or at slaughter (LT). A decrease (p<0.10) in intramuscular omega-3 fatty acids content contributed by the decrease in α-linolenic acid was observed in HRUP group both during the growing stage (LM) and at slaughter (LT).

### Effects of the RUP level on relative mRNA expression of lipid metabolic genes in muscle biopsy tissue and carcass longissimus muscle

**Figure 1:**
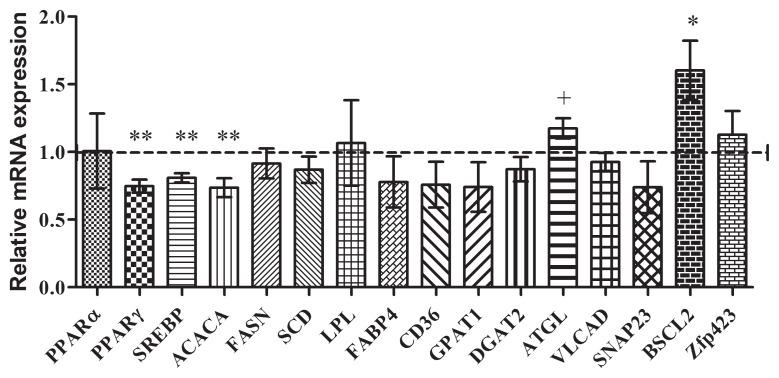
Effect of increasing the dietary RUP:RDP ratio on the relative mRNA expression levels of genes in the intramuscular tissue of Hanwoo steers at 17 months of age (biopsy). Values are least-square means (n = 16 per group), with standard errors affixed to the bars, expressed as expression relative to the control. ^†^ p≤0.1, * p<0.05, ** p<0.01. RUP, rumen undegradable protein; RDP, rumen degradable protein.

The relative mRNA expression levels of key genes involved in fat metabolism during the growing stage were significantly different between the dietary treatments. The genes peroxisome proliferator-activated receptor γ (PPARγ), sterol regulatory element-binding protein (SREBP), and acetyl-CoA carboxylase (ACACA) were downregulated (p<0.05) in the HRUP group, suggesting lower fat biosynthesis activity (Figure 1). In contrast, adipose triglyceride lipase (ATGL) (p = 0.087) and Berardinelli-Seip congenital lipodystrophy 2-seipin (BSCL2) (p<0.05), which are involved in lipid breakdown and regulation, were upregulated in the HRUP group.

**Figure 2:**
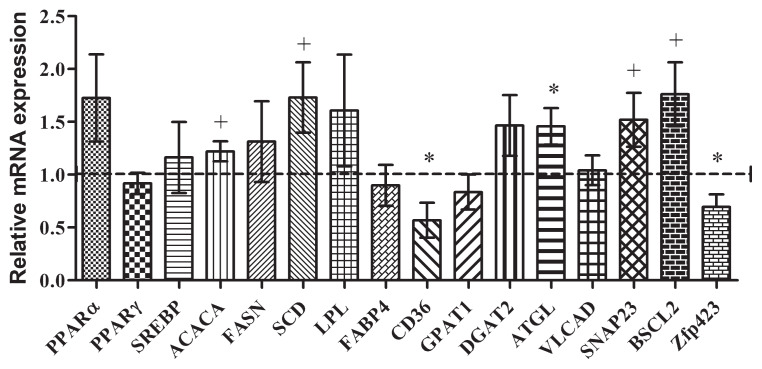
Effect of increasing the RUP ratio on the relative mRNA expression levels of genes in the intramuscular tissue of Hanwoo steers at 26 months of age (slaughter). Values are least-square means (n = 16 per group), with standard errors affixed to the bars, expressed as expression relative to the control. ^†^ p≤0.1, * p<0.05. RUP, rumen undegradable protein.

A trend toward upregulation of stearoyl-CoA desaturase (p = 0.063) and ACACA (p = 0.087) was observed at slaughter in the HRUP group, indicating a potential increase in fatty acid synthesis. In contrast, the relative mRNA expression levels of synaptosome-associated protein 23 (SNAP23) (p = 0.063), ATGL (p<0.05), BSCL2 (p = 0.087), fatty acid translocase (CD36) (p<0.05), and zinc finger protein 423 (Zfp423) (p<0.05) were downregulated in the HRUP group, suggesting altered fat mobilization and lipid metabolism (Figure 2).

## DISCUSSION

Shortening the feeding period of Hanwoo steers to 26 months, compared to the conventional 30 months, reduces environmental pollution and production costs. However, achieving this without compromising animal performance is crucial. Although the concentrate treatments were designed with a target of 18% CP on a DM basis for the fattening stage, the LRUP concentrate had a higher CP content (20% DM). This discrepancy, which was likely caused by unexpected variations in the moisture and nutrient contents of the ingredients, which can fluctuate depending on factors such as seasonal changes, storage conditions, variations in raw material sourcing and differences between production batches. These inconsistencies may have altered the overall nutrient balance of the formulated diet, leading to a higher-than-intended CP concentration in the LRUP treatment during the fattening stage, and low neutral detergent fiber (NDF) and high ether extract (EE) content in HRUP group during the growing stage. Such deviations, while unplanned, emphasize the challenges of maintaining consistent nutrient levels in practical feed formulations. While a decrease in dietary CP intake has been linked to negative effects on growth performance in beef cattle [15], the HRUP group, despite consuming 0.2 kg less CP per day, demonstrated higher total weight gain, ADG, and FCR during the fattening phase compared to the LRUP. This improvement was likely due to the increased intake of RUP, which enhanced N retention, N utilization, and the supply of metabolizable protein in growing beef cattle, thereby increasing their growth efficiency [16]. In support of this contention, analyses of the rumen NH_3_-N concentration during the growing stage indicated that the concentration in the HRUP group was 23% lower than in the LRUP group, suggesting reduced protein degradation in the rumen and greater N availability for the animals.

The inadequate ruminal NH_3_-N available for fermentation and microbial synthesis caused by a low RDP can lead to lower DMI due to lower activity of rumen microorganisms [17]. However, no such negative effects were observed in this study, likely due to the availability of an optimal NH_3_-N concentration necessary for microbial protein synthesis [18]. Similar results were observed in previous studies where differences in the dietary RDP level did not affect the DMI of Hanwoo steers [6,11]. Additionally, ruminants fed high RDP are expected to have higher BUN concentrations than those fed high-RUP diets due to the higher deamination of amino acids in the rumen and subsequent absorption of N into the bloodstream [19]. However, in this study, BUN levels were similar between the LRUP and HRUP groups, consistent with a previous study [12]. This finding suggests that the capacity of the ruminal microorganisms to efficiently utilize available ruminal NH_3_-N was high during the growing stage of Hanwoo steers, regardless of differences in the RUP:RDP ratio in the diet.

Lower acetate levels have been reported when animals are fed high-RUP diets [8], likely due to increased inclusion of FSBM. Earlier studies have reported that FSBM suppressed abundance of cellulolytic bacteria such as *Ruminococcus albus* and *Butyrivibrio fibrisolvens* [8] that are involved in acetate production. Studies have also suggested that the supplemental RUP improved efficiency of nutrient and acetate utilization in growing lambs that led to lower acetate levels in the rumen [20]. The higher ruminal acetate content, and consequently the higher acetate-to-propionate ratio in the LRUP diet, may partly explain the decrease in the ADG. Notably, propionate and butyrate provide more energy in ruminants than acetate from a stoichiometric perspective, which influences gain and feed efficiency [21]. Conversely, the higher proportion of iso-valerate in the rumen of animals in the HRUP group may have further supported MP synthesis, as iso-valerate is a key substrate and an indicator of the efficiency of MP synthesis [22]. Although a direct evaluation of rumen MP production and intestinal N availability is necessary, the increase in RUP proportion via FSBM suggests a better balance between MP synthesis and the amount of CP reaching the small intestine in the HRUP group. This balanced protein metabolism was particularly evident during the growing phase of Hanwoo steers, when the rate of protein deposition was high, suggesting that N from RUP was efficiently incorporated into tissue, supporting muscle growth and leading to increased BW gains. The positive effects of high protein availability on BW gains align with previous studies conducted with Hanwoo steers [6] but contrast with findings from Bharanidharan et al [5] and Jeon et al [12]. Notably, a high CP diet was supplied during the middle or late fattening stages of Hanwoo steers in the latter studies, unlike in the current study. This suggests that precision nutritional strategies corresponding to the growth stage should be considered when implementing strategies to enhance the performance of Hanwoo steers.

The increase in the final live weight of the Hanwoo steers fed the HRUP diet in this study did not translate to a significant increase in carcass weight (CW). Although further investigations are needed, the probable increased intestinal availability of CP may have enhanced bone weight and density [23], which likely contributed to greater BW gain during the later stages of growth without affecting CW. Previous studies have suggested that CW is directly related to the rib eye area in Hanwoo cattle [24]. Although no group differences were observed in CW in the current study, a minor increase in rib eye area highlighted the importance of RUP and N availability during the growing period of Hanwoo steers. Similar results were reported by an earlier study, which found higher amino acids in the duodenum of crossbred beef steers fed heat-treated SBM with high RUP content, leading to a tendency for increased rib eye area in the longissimus muscle [25]. However, despite the increased N availability, there was no effect on marbling or other carcass traits in our study.

IMF content is a key factor in determining MS in beef cattle, and the lack of variation in the IMF content or fatty acid profile aligns with the absence of differences in MS observed in this experiment. These results contradict previous findings, such as those of Faucitano et al [25], who reported higher IMF and MS in beef steers fed lignosulfonate-treated SBM rich in RUP. Similarly, a previous study reported that higher RUP concentrations (6.0% vs. 6.7% DMI) in finishing lamb diets increase IMF content [26], while Lee et al [11] reported higher MS in Hanwoo steers fed high-RUP concentrate (4.2% vs. 5.1% DMI). However, Hanwoo steers in the study by Lee et al [11] were slaughtered at an average age of 30 months. As the accumulation of IMF accelerates significantly between 23 and 30 months [27], slaughtering the animals at 26 months in the present study may have limited the effects of increased N availability on IMF accumulation and MS improvements. This was further supported by the relatively lower IMF content (15%) in the current study compared to our previous study (17%), where Hanwoo steers were slaughtered at 30 months of age [3]. Total CP intake relative to the RUP levels during the growing and fattening stages may also have contributed to the inconsistency. A previous study showed that as the RUP levels rose in high-CP diets (above 14.5%), MS tended to decline [28]. In contrast, a previous Hanwoo study [11] maintained CP levels below 15%, while in the current study the levels exceeded 20% DMI during the growing stage. In support of this, intramuscular mRNA expression of key adipogenesis and fat synthesis transcription factors, such as PPARγ and SREBP, and genes like ACACA, were downregulated in biopsy samples collected during the growing stage from the HRUP group [29–31]. Interestingly, in carcass samples, genes such as ACACA, BSCL2, and SNAP23, which increase fat synthesis and droplet size [32,33], were upregulated, indicating a compensatory mechanism that mitigated the anticipated effects of high RUP intake on IMF and MS. Furthermore, upregulation of ATGL, a gene involved in the early stages of lipolysis within adipocytes [34], suggests a balance between lipid synthesis and oxidation during the late fattening stages in the HRUP group. However, a decline in omega-3 fatty acids, primarily α-linolenic acid, was observed in both biopsy and slaughter samples from the HRUP group. Given that omega-3 fatty acids regulate adipose tissue metabolism and energy pathways through PPARγ expression [35], this reduction may have influenced metabolic processes. During the growing stage, when adipose tissue is relatively less abundant, the decrease in omega-3 fatty acid content coincided with lower PPARγ expression. However, during the fattening stage, when adipose tissue accumulation is more pronounced, no significant differences in PPARγ expression were observed, suggesting that omega-3 fatty acid levels may not have had a substantial impact on adipose tissue metabolism. On the other hand, omega-3 fatty acids are also known to regulate metabolic processes in muscle tissue [36]. In this study, ATGL expression remained elevated in the HRUP group during both growing stage and at slaughter, indicating a potential shift toward β-oxidation for energy production. This metabolic adaptation may have contributed to the lower omega-3 content in meat while supporting muscle synthesis, which could partially explain the improved rib-eye area observed in the HRUP group.

Overall, reducing the slaughter age of Hanwoo steers to 26 months, while improving the RUP supply, did not negatively affect the performance or carcass characteristics of the steers. The final BW achieved by HRUP at 26 months, despite a relatively lower CP intake during the fattening stage, was comparable to that of animals raised on the conventional 30-month fattening program [37,38]. However, the relatively lower IMF and omega-3 fatty acid content, could be a drawback, as it may affect the nutritional aspect, tenderness and other sensory traits of the meat. The absence of significant positive effects of high RUP intake on carcass characteristics, particularly IMF accumulation, may have been influenced by unexpected variations in total CP intake during the fattening stage, and also due to the lower NDF and higher EE intake in HRUP during the growing stage. However, the absence of differences in serum glucose, NEFA and triglyceride levels suggests that these effects were minimal. This highlights the need for further research to identify the optimal CP and RUP levels in iso-nutrient feed formulations to better understand fat metabolism and deposition while also maintaining overall performance in a shortened feeding period.

## CONCLUSION

In this study, the RUP content in the concentrate was increased by approximately 10% to shorten the fattening period to 26 months. This enhancement in RUP positively influenced protein metabolism by meeting microbial protein requirements and increasing the flow of protein to the small intestine, ultimately improving protein efficiency. Consequently, notable improvements were observed in daily weight gain, FCR, and rib-eye area, attributed to the greater availability of essential amino acids critical for muscle synthesis. Therefore, implementing a 26-month fattening program, facilitated by increased RUP intake from the growing stage onward, is anticipated to enhance productivity while also reducing feed costs and minimizing environmental impact. However, further investigation is required to accurately determine the optimal RUP:RDP ratio for different dietary CP levels at different growth stages of Hanwoo beef cattle.

## Figures and Tables

**Table 1:** Ingredient composition of the concentrates fed to the animals

Ingredients (% DM)	Growing stage^1)^		Fattening stage^2)^	
	
	LRUP^3)^	HRUP	LRUP	HRUP
Corn flaked	-		25.0	30.1
Wheat fine	14.0	12.5	17.0	15.0
Corn gluten feed	14.0	16.4	15.5	6.7
Fermented soybean meal^4)^	-	4.0	-	2.5
Soybean meal	6.7	-	-	-
Corn DDGS	15.0	22.4	12.3	17.2
Corn fine	10.5	13.7	8.0	2.0
Soy hulls	11.5	3.2	-	-
Oat straw ground	-	-	7.0	-
Molasses mix	5.0	1.8	4.5	2.0
Palm kernel meal	13.0	16.0	2.4	14.0
Rice bran	2.5	5.5	2.0	3.0
Corn cobs	-	-	2.0	-
Limestone	3.0	3.4	1.3	2.8
Alfalfa pellet	4.0	-	-	-
Bentonite	-	-	1.0	-
Buffer mix	-	-	0.8	0.6
Urea	-	-	0.6	-
Salt	0.4	1.0	0.2	0.3
Mineral premix	0.3	0.1	0.2	0.1
Ammonium chloride	-	-	0.2	0.1
Vitamin premix	0.1	0.0	0.1	0.1
Molasses	-	-	-	1.0
Yeast fermentation product	-	-	-	0.1
CMS lysine by product	-	-	-	1.9
Corn small coarse	-	-	-	0.6

1) Growing stage, from 8 to 17 mo of age.

2) Fattening stage, from 18 to 26 mo of age.

3) LRUP, low RUP:RDP concentrate (37:63 of CP); HRUP, high RUP:RDP concentrate (47:53 of CP).

4) SoELAB-Pass, FEEDUP Ltd., Korea.

DDGS, distiller’s dried grains with soluble; CMS, condensed molasses soluble; RDP, rumen degradable protein.

**Table 2:** Chemical composition of the experimental diet

Composition (DM basis)	Growing stage^1)^			Fattening stage^2)^		
	
	Timothy hay	LRUP^3)^	HRUP	Rice straw hay	LRUP	HRUP
OM (%)	88.1	88.4	87.4	84.2	86.5	87.1
CP (%)	4.5	20.4	21.0	3.8	20.1	17.8
Ether extract (%)	0.6	5.8	6.9	0.9	3.7	5.3
Ash (%)	4.0	7.8	9.6	11.4	9.5	8.3
NDF (%)	69.4	34.5	30.7	65.2	25.3	24.2
ADF (%)	42.4	15.5	13.5	38.8	9.7	11.5
RDP (% of CP)	49.9	62.8	53.8	16.8	63.0	53.0
RDP (%)	2.3	12.8	11.3	0.6	12.7	9.4
RUP (% of CP)	50.1	37.2	46.2	83.2	37.0	47.0
RUP (%)	2.3	7.6	9.7	3.1	7.5	8.4
TDN (%)	58.2	72.0	72.0	60.7	74.0	74.0

1) Growing stage, from 8 to 17 mo of age.

2) Fattening stage, from 18 to 26 mo of age.

3) LRUP, low RUP:RDP concentrate (37:63 of CP); HRUP, high RUP:RDP concentrate (47:53 of CP).

OM, organic matter; CP, crude protein; NDF, neutral detergent fiber; ADF, acid detergent fiber; RUP, rumen undegradable protein; RDP, rumen degradable protein; TDN, total digestible nutrients.

**Table 3:** Effects of dietary treatments on intake and growth performance of Hanwoo steers (n = 3^2)^

Item	LRUP^1)^	HRUP	SEM	p-value
Body weight (kg)				
Initial	217.1	217.1	12.84	0.996
Growing stage	491.6	498.7	14.74	0.649
Fattening stage (Final)	714.4	744.0	17.40	0.140
Total weight gain	497.3	526.9	7.34	0.007
Intake (kg/d DM)				
Growing stage^2)^				
Timothy hay	3.22	3.21	0.07	0.829
Concentrate	4.62	4.60	0.12	0.284
Total CP	1.09	1.11	0.01	0.284
RUP	0.42	0.52	0.01	<0.0001
RDP	0.67	0.59	0.01	<0.0001
Fattening stage^3)^				
Rice straw hay	0.93	0.97	0.03	0.165
Concentrate	8.23	8.28	0.12	0.738
Total CP	1.69	1.51	0.02	<0.001
RUP	0.64	0.72	0.01	<0.001
RDP	1.05	0.79	0.01	<0.0001
Average daily gain (kg/day)				
Growing stage	1.00	1.03	0.03	0.289
Fattening stage	0.81	0.88	0.02	0.004
Entire period^4)^	0.90	0.96	0.01	0.006
Feed conversion ratio^5)^				
Growing stage	7.90	7.61	0.19	0.173
Fattening stage	11.37	10.49	0.25	0.013
Entire period	9.79	9.25	0.14	0.008

1) LRUP, low RUP:RDP concentrate (37:63 of CP); HRUP, high RUP:RDP concentrate (47:53 of CP).

2) Growing stage, from 8 to 17 mo of age.

3) Fattening stage, from 18 to 26 mo of age.

4) Entire period, from 8 to 26 mo of age.

5) Feed conversion ratio = average daily DM intake / average daily gain.

SEM, standard error of the mean; DM, dry matter; CP, crude protein; RUP, rumen undegradable protein; RDP, rumen degradable protein.

**Table 4:** Effects of the dietary treatments on carcass and marbling characteristics of Hanwoo steers slaughtered at 26 months of age (n = 3^2)^

	LRUP^1)^	HRUP	SEM	p-value
Yield grade traits				
Carcass weight (kg)	418.1	426.3	8.8	0.387
Dressing percentage^2)^ (%)	59.4	58.3	0.6	0.085
Yield grade (A: B: C, head)	10: 05: 01	06: 08: 02		
Yield grade score^3)^	2.5	2.3	0.27	0.390
Rib eye area (cm^2^)	81.5	83.9	1.14	0.081
Back fat thickness (mm)	12.1	11.0	1.90	0.596
Yield index^4)^	62.0	62.2	0.63	0.712
Quality grade traits				
Quality grade (1^++^: 1^+^: 1: 2, head)	4: 1: 7: 4	1: 3: 10: 2		
Marbling score^5)^	5.0	4.8	0.58	0.758
Image analysis				
Marbling percentage	0.2	0.2	0.02	0.829
Number of marbling particles	3,741.0	2,666.0	734.0	0.193
Number of coarse marbling particle	61.8	63.4	7.15	0.828
Marbling area (cm^2^)	16.0	16.3	1.57	0.838
Number of fine marbling particle	186.8	190.4	13.7	0.800
Fineness of marbling	2.3	2.3	0.15	0.735

1) LRUP, low RUP:RDP concentrate (37:63 of CP); HRUP, high RUP:RDP concentrate (47:53 of CP).

2) Dressing percentage = carcass weight / shrunk weight.

3) A, 3; B, 2; C, 1.

4) Yield index value = {11.06398–1.25149×Back fat thickness (mm)+0.031805×Rib eye area (cm^2^)+0.54952×Carcass weight (kg)×100}.

5) Marling score (quality grade): 9–7 (1^++^), 6 (1^+^), 5–4 (^1)^, 3 –2 (^2)^, 1 (^3)^.

SEM, standard error of the mean; RUP, rumen undegradable protein; RDP, rumen degradable protein; CP, crude protein.

**Table 5:** Effects of dietary treatments on rumen fermentation characteristics of Hanwoo steers during the growing stage (n = 32)

Item	LRUP^1)^	HRUP	SEM	p-value
pH	6.3	6.0	0.16	0.126
NH_3_-N (mg/dL)	22.0	17.0	2.18	0.062
Total VFA (mM)	102.2	95.2	3.80	0.116
VFAs (mM)				
Acetate	64.6	58.1	2.48	0.040
Propionate	19.4	19.6	0.79	0.767
Iso-butyrate	0.9	0.9	0.03	0.135
Butyrate	14.0	13.5	0.58	0.438
Iso-valerate	1.2	1.4	0.97	0.097
Valerate	2.1	1.7	0.13	0.027
A:P ratio	3.3	3.0	0.05	0.001

1) LRUP, low RUP:RDP concentrate (37:63 of CP); HRUP, high RUP:RDP concentrate (47:53 of CP).

SEM, standard error of the mean; RUP, rumen undegradable protein; RDP, rumen degradable protein; VFA, volatile fatty acid; A:P ratio, acetate to propionate ratio; CP, crude protein.

**Table 6:** Effects of dietary treatments on blood metabolites of Hanwoo steers during the growing stage (n = 32)

Item	LRUP^1)^	HRUP	SEM	p-value
Total protein (g/dL)	7.3	7.3	0.11	0.955
Albumin (g/dL)	3.8	3.9	0.07	0.558
Creatinine (mg/dL)	1.3	1.4	0.06	0.242
Blood urea nitrogen (mg/dL)	20.3	20.5	0.78	0.770
AST (U/L)	66.7	72.7	4.09	0.193
ALP (U/L)	142.4	159.6	25.62	0.526
GGT (U/L)	16.4	24.0	4.43	0.141
Glucose (mg/dL)	72.2	74.7	2.15	0.290
NEFA (mmol/L)	0.2	0.2	0.02	0.561
β-Hydroxybutyrate (mmol/L)	0.6	0.6	0.06	0.918
Total-cholesterol (mg/dL)	168.6	172.4	15.36	0.812
Triglycerides (mg/dL)	18.5	18.8	1.29	0.788

1) LRUP, low RUP:RDP concentrate (37:63 of CP); HRUP, high RUP:RDP concentrate (47:53 of CP).

SEM, standard error of the mean; AST, aspartate aminotransferase; ALP, alkaline-phosphatase; GGT, gamma-glutamyl transferase; NEFA, non-esterified fatty acid; RUP, rumen undegradable protein; RDP, rumen degradable protein; CP, crude protein.

**Table 7:** Effects of dietary treatment on fatty acid composition of biopsied *longissimus lumborum* (LM) muscle in Hanwoo steers during the growing stage (17 months of age; n = 16)

Fatty acid (mg/100 g FAME)	LRUP^1)^	HRUP	SEM	p-value
Myristic acid (C14:0)	2,727	3,195	336.1	0.213
Palmitic acid (C16:0)	23,986	24,684	926.1	0.479
Palmitoleic acid (C16:^1)^	4173	4208	562.8	0.952
Stearic acid (C18:0)	9915	9728	961.2	0.852
Elaidic acid (C18:1n9t)	1372	1301	103.7	0.521
Oleic acid (C18:1n9c)	36,340	34,726	1,499.4	0.323
Linoleic acid (C18:2n6c)	3,041	2,845	383.5	0.628
α-Linolenic acid (C18:3n^3)^	169	133	35.8	0.031
Others^2)^	4,130	4,071	217.5	0.797
SFA^3)^	38,748	39,638	1,706.2	0.621
MUFA^4)^	43,384	41,786	2,029.1	0.461
PUFA^5)^	3,720	3,469	444.6	0.593
Omega-6	3,514	3,307	431.9	0.649
Omega-3	206	162	16.8	0.038
Total fatty acids (mg/100 g FAME)	85,852	84,893	2,530.1	0.718

1) LRUP, low RUP:RDP concentrate (37:63 of CP); HRUP, high RUP:RDP concentrate (47:53 of CP).

2) C4:0+C6:0+C8:0+C10:0+C11:0+C12:0+C13:0+C14:1+C15:0+C15:1+C17:0+C17:1+C18:2n6t+C20:0+C18:3n6+C20:1n9+C21:0+C20:2+C22:0+C20:3n6+ C22:1n9+C20:3n3+C23:0+C20:4n6+C22:2+C24:0+C20:5n3+C24:1n9+C22:6n3.

3) C10:0+C11:0+C12:0+C14:0+C15:0+C16:0+C17:0+C18:0+C20:0+C21:0 +C22:0+C24:0.

4) C14:1n5+C16:1n7+C17:1n7+C18:1n7+C18:1n9+C20:1n9+C22:1n9+C24:1n9.

5) C18:2n6+C18:2c9,t11+C18:3n3+C18:3n6+C20:2n6+C20:3n3+C20:3n6+C20:4n6+C20:5n3+C22:2n6+C22:4n6+C22:5n3+C22:6n3.

FAME, fatty acid methyl esters; SEM, standard error of the mean; SFA, saturated fatty acids; MUFA, monounsaturated fatty acids; PUFA, polyunsaturated fatty acids; RUP, rumen undegradable protein; RDP, rumen degradable protein.

**Table 8:** Effects of dietary treatments on fatty acid composition of *longissimus thoracis* (LT) muscle in Hanwoo steers slaughtered at 26 months of age (n = 3^2)^

Fatty acid (mg/100 g meat)	LRUP^1)^	HRUP	SEM	p-value
Myristic acid (C14:0)	469	456	122.3	0.916
Myristoleic acid (C14:^1)^	144	116	26.9	0.337
Palmitic acid (C16:0)	3,351	3,294	679.7	0.936
Palmitoleic acid (C16:^1)^	668	593	127.4	0.576
Stearic acid (C18:0)	1,168	1,199	222.5	0.895
Elaidic acid (C18:1n9t)	164	203	49.5	0.456
Oleic acid (C18:1n9c)	5,140	5,351	945.4	0.831
Linoleic acid (C18:2n6c)	286	292	37.0	0.880
α-linolenic acid (C18:3n^3)^	15	8	3.1	0.068
Others^2)^	327	357	54.7	0.598
SFA^3)^	5,182	5160	1,046.8	0.984
MUFA^4)^	6,216	6,,375	1,124.6	0.892
PUFA^5)^	336	335	43.6	0.983
Omega-6	321	326	41.2	0.890
Omega-3	15	8	3.1	0.068
Intramuscular lipid (%)	15	15	2.7	0.842
Total fatty acids (mg/100 g Meat)	1,1734	1,1870	2,172.6	0.952

1) LRUP, low RUP:RDP concentrate (37:63 of CP); HRUP, high RUP:RDP concentrate (47:53 of CP).

2) Others= C4:0+C6:0+C8:0+C10:0+C11:0+C12:0+C13:0+C15:0+C15:1+C17:0+C17:1+C18:2n6t+C20:0+C18:3n6+C20:1n9+C21:0+C20:2+C22:0+C20:3n6+ C22:1n9+C20:3n3+C23:0+C20:4n6+C22:2+C24:0+C20:5n3+C24:1n9+C22:6n3.

3) C10:0+C11:0+C12:0+C14:0+C15:0+C16:0+C17:0+C18:0+C20:0+C21:0 +C22:0+C24:0.

4) C14:1n5+C16:1n7+C17:1n7+C18:1n7+C18:1n9+C20:1n9+C22:1n9+C24:1n9.

5) C18:2n6+C18:2c9,t11+C18:3n3+C18:3n6+C20:2n6+C20:3n3+C20:3n6+C20:4n6+C20:5n3+C22:2n6+C22:4n6+C22:5n3+C22:6n3.

SEM, standard error of the mean; SFA, saturated fatty acids; MUFA, monounsaturated fatty acids; PUFA, polyunsaturated fatty acids; RUP, rumen undegradable protein; RDP, rumen degradable protein; CP, crude protein.
